# Animal personality in multiple stressor environments: the evolutionary ecology of among-individual differences in responses to stressor suites

**DOI:** 10.1098/rspb.2024.1620

**Published:** 2024-10-23

**Authors:** Andrea S. Grunst, Melissa L. Grunst

**Affiliations:** ^1^ Department of Biology, Indiana State University, Terre Haute, IN 47809, USA

**Keywords:** animal personality, behavioural syndromes, multiple stressors, trade-offs, anthropogenic environmental change, urban ecology

## Abstract

Animal personality differences may have evolved as alternative strategies for negotiating multiple stressor landscapes. Indeed, ecologists are increasingly recognizing that interactions among multiple stressors can transform selective landscapes and behavioural and physiological responses to stress regimes. Yet, evaluating this hypothesis poses challenges, as most studies involving relationships between personality variation and the environment consider single stressors. Here, we review the literature to explore the theory and evidence that multiple stressor environments may mediate personality variation. We consider effects on evolution of personality variation, as influenced by life-history, energetic and behavioural trade-offs, and effects on phenotypic expression of personality traits. We then explore how personality variation may modulate behavioural and physiological responses to multiple stressors, and how differential responses may be affected by personality-dependent movement ecology and cognitive strategies. Among-individual differences in responses to multiple stressors are critical to elucidate, as multi-stress interactions may transform animal behavioural and physiological responses relative to those predicted under single stressor scenarios, and because among-individual variation comprises the basis for evolutionary shifts in stress responsiveness and population resiliency to global environmental change.

## 1. Introduction

Animal personality is defined by consistent among-individual differences in behaviour across time and contexts, and can be key to understanding among-individual variation in responses to environmental stressors. Personality traits often form suites of co-adapted behavioural phenotypes, termed behavioural syndromes. For example, individuals that are bolder towards predators are often less neophobic, more aggressive and highly active [1]. Limits to behavioural plasticity, fluctuating selection, frequency-dependent selection [1,2] and alternatives for balancing life-history trade-offs and managing pace-of-life [3,4] have been proposed to promote personality evolution. Moreover, personality variation may be associated with stress-coping styles, with bold-aggressive (proactive) individuals responding to stressors actively and aggressively (fight-flight and sympathetic response), and shy (reactive) individuals responding more passively (freeze-hide, parasympathetic and hypothalamus–pituitary–adrenal (HPA) response) [5]. Thus, alternatives for coping with multi-stress environments might be central to explaining personality evolution. Indeed, personality variation has been extensively studied in the context of responses to fundamental ecological stressors, such as predation risk. For instance, bold-aggressive behavioural syndromes in three-spined sticklebacks (*Gasterosteus aculeatus*) exist only in high predation populations [6], and emerge following experimental predator exposure [7].

A growing literature suggests that multiple stressor interactions can be critical to predicting individual-, population- and ecosystem-level ramifications of stress regimes [8]. Multi-stress studies have documented that stressors can combine additively, synergistically or antagonistically to affect biological outcomes, from individual behaviour to ecosystem function [8]. For example, increased temperature amplified suppressive effects of predation risk on prey foraging behaviour in a rocky shore system, resulting in negative growth efficiency [9]. Furthermore, a recent review addressed how behaviour might be integrated into multiple stressor frameworks [10]. This review highlighted that behavioural plasticity comprises a critical mechanism by which animals adjust to multi-stress landscapes, and that behaviour likely shapes stressor suites to which animals are exposed [10]. Yet, relatively few papers regarding animal personality variation have adopted a multi-stress perspective.

Here, we review the idea that interplay among multiple stressors may be central to understanding evolution and phenotypic expression of animal personality and among-individual differences in responses to multiple stressors, including those associated with anthropogenic environmental change. In some cases, one predominant stressor might drive personality evolution, for instance in top-down controlled populations with intense predation [6]. However, in many, we predict that multiple stressors may combine to determine selective landscapes acting on behavioural traits and personality variation.

## 2. Review objectives and structure

**Figure 1.**
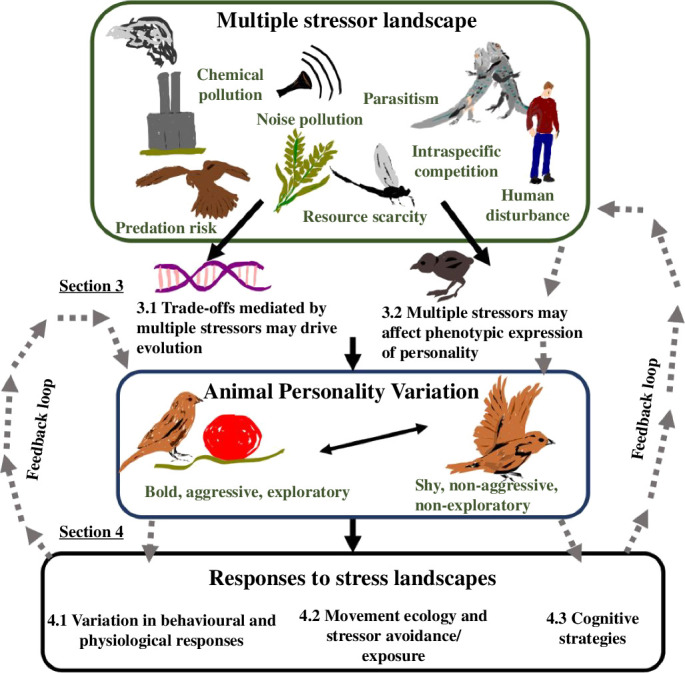
Framework for understanding personality variation in multi-stress environments, and paper outline. Multiple stressors may affect (§3.1) personality evolution and (§3.2) phenotypic expression of personality. In turn, personality may affect responses to multiple stressors, with effects mediated by (§4.1) among-individual differences in behavioural and physiological responses, (§4.2) differential avoidance/exposure, mediated by movement ecology and (§4.3) cognitive strategies. Circular pathways demarcated by dashed arrows indicate the potential for feedback dynamics, wherein personality-dependent responses to multiple stressors may feedback to modify both personality trait expression and stressor suites to which individuals are exposed. Note that either positive or negative feedback effects may be possible.

In this review (methodology in electronic supplementary material), we evaluate empirical evidence and theory addressing two interrelated questions. First, do multi-stress landscapes promote personality variation (§3), and second, does personality affect responses to shifting multi-stress regimes (§4)? In §3, we consider personality variation caused by evolution, especially as mediated by trade-offs arising from negotiating multi-stress landscapes, and phenotypic variation, determined by behavioural reaction norms, developmental effects and state dependency. In §4, we consider how personality may affect multi-stress responses by mediating among-individual differences in behavioural and physiological responses, movement ecology and cognitive strategies. We conclude with future research priorities (§5). Figure 1 presents an outline for our paper and framework for understanding personality variation in multi-stress landscapes. See electronic supplementary material, table S1 for a non-exhaustive list of studies identified in our review involving effects of multi-stress environments on personality variation (electronic supplementary material, table S1*a*), and differential stressor responses by personality types (electronic supplementary material, table S1*b*). Studies explicitly investigating multi-stress effects on personality are rare. Hence, we also integrated studies that indirectly suggest hypotheses regarding how multiple stressors may affect personality.

## 3. Do multi-stress landscapes promote personality variation?

### (a) Trade-offs mediated by multiple stressors may drive personality evolution

Differential balance of survival-reproduction trade-offs, mediated by interplay between stressors such as predation and resource limitation, has been hypothesized to underlie personality evolution [3,4]. Pace-of-life theory suggests that intercorrelated behavioural and physiological traits determine energy allocation, with bold-aggressive personality types prioritizing reproduction over survivorship [3]. However, evidence that life-history trade-offs underpin personality variation is mixed. A meta-analysis using unpartitioned phenotypic variance found that bolder animals exhibit faster reproduction and lower survivorship, but fitness correlates of aggression were unclear [11]. Other meta-analyses and reviews question the relationship between survival-reproduction trade-offs and personality [12–15].

One meta-analysis found no overall directional relationship between risk-taking and mortality, and riskier behaviour counterintuitively correlated with higher survivorship in the wild [13]. Another suggested that resource acquisition predominates over allocation in determining correlations between behaviour and fitness [14], as may occur given state-dependent personality and best-of-a-bad-job strategies [16].

This equivocal evidence suggests that if life-history trade-offs and pace-of-life syndromes (POLSs) contribute to personality evolution, they may emerge only in specific contexts. Indeed, a POLS was found in urban, but not rural, *Daphnia magna* genotypes [17]. Similarly, in lemon sharks (*Negaprion brevirostris*), open field exploration correlated with riskier foraging behaviour and a growth-mortality trade-off in low, but not higher predation context [18]. The combination and intensity of multiple stressors may determine viable behavioural alternativeness for balancing life-history trade-offs, and whether POLSs and personality variation arise (electronic supplementary material, figure S1*a*), but evidence remains inconclusive.

Furthermore, in multi-stress environments, personality variation might reflect behavioural trade-offs and alternative strategies for coping with multiple stressors, without variation in the balance of life-history trade-offs necessarily emerging. For instance, much evidence suggests that boldness may increase predation risk, as in radio-tagged roach (*Rutilus rutilus*) depredated by cormorants (*Phalacrocorax carbo*) [19]. However, bolder behaviour might not always translate into higher mortality, if bolder individuals better evade predators [16], or if different stress coping strategies lead to different mortality sources (electronic supplementary material, figure S1*b*). Below we evaluate ways in which trade-offs and other interactions among multi-stress combinations may affect personality evolution.

#### (i) Predation and resources

Predation has received the most attention from researchers interested in stressor effects on personality. Bold-aggressive behavioural syndromes can emerge in predation-impacted populations, as seminally demonstrated in sticklebacks [6,7]. Predation may induce personality variation and behavioural syndromes by generating a trade-off between acquiring resources versus avoiding predation [19]. In this case, resource stress and predation might combine to determine personality trait means and (co)variances. Indeed, a state-dependent model suggested that personality variation should be greatest at intermediate combinations of predation and resources [20]. In contrast, under highly favourable (low risk and high resources) or unfavourable (high risk and low resources) conditions, bold or shy personality predominated, respectively [20]. In this model, individuals in better initial state remained bolder over time under intermediate conditions. Intermediate conditions could also promote genetic personality variation by generating alternatives for negotiating multi-stress landscapes, as suggested by modelling studies not founded on state dependence [21].

Some empirical evidence also suggests that resource availability may combine with predation to shape behavioural trade-offs and selection on personality, but evidence remains limited and indirect. For instance, in convict cichlids (*Amatitlania nigrofasciata*), high predation and low resources additively increased neophobia towards a novel chemical. Resource-limited fish may not have time to learn to identify predators, favouring increased neophobia [22]. Conversely, other studies report that resource limitation increases risk-taking [23], suggesting that resource limitation might antagonize suppressive effects of predators on boldness. Some studies also suggest that resource availability does not modulate predation’s effects on personality traits. For example, predation risk reduced activity and boldness in pond water striders (*Gerris marginatus*), but resource supply had null effects [24]. As outlined in electronic supplementary material, figure S2, how, and whether, resource limitation combines with predation risk to affect the selection on personality variation might oftentimes depend on the extent to which proactive behaviour accentuates costs of resource limitation through increased metabolic demand (electronic supplementary material, figure S2*a*), versus ameliorates impacts through increased resource-holding capacity (electronic supplementary material, figure S2*b*), but this hypothesis remains to be tested.

In addition, selective dynamics are likely to be complicated by additional co-stressors. Indeed, multi-stress interactions often involve more than two stressors, complicating eco-evolutionary outcomes [8]. For instance, metabolic expenses of proactive behaviour could reduce energy available to combat parasitism, especially in highly resource-limited environments, but enhanced resource-holding capacity could alternatively increase energy available for immune defences [25]. Below, we further explore how additional co-stressors may mediate multi-stress effects that modulate behavioural trade-offs and selection on personality.

#### (ii) Competition

Intra- and interspecific competition are key ecological stressors that may affect behavioural trade-offs within multi-stress environments, modifying selection on personality. First, social stress associated with intraspecific competition may trade-off against benefits of resource acquisition. For instance, in great tits (*Parus major*), viability selection favoured fast exploration, which is linked to aggression, under low conspecific density, but slow exploration under high density, with fluctuating selection across population cycles [26]. Hence, the costs of social stress associated with aggression may outweigh benefits when conspecific density becomes too high [26]. On the other hand, bold-aggressive behaviour may be favoured during periods of intense competition if benefits of resource acquisition capacity outweigh costs. For instance, less stress-reactive mice (*Mastomys natalensis*) took more risks and had higher survivorship during population decline phases, when competition was intense [27].

Competition may also combine with predation to influence selection on personality. For instance, in larval damselflies (*Ischnura verticalis*), intraspecific aggression is conspicuous to predators [28]. Thus, predation and competition might interact to select against aggression. Alternatively, aggressiveness may decrease predation by allowing refuge monopolization, such that predation and competition might interactively favour aggression, potentially independent of boldness. For example, less aggressive damelfishes (*Dascyllus* sp.) were displaced from shelters and suffered elevated predation [29]. In addition, male lava lizards (*Tropidurus hispidus*) re-emerged sooner when predator attack occurred after aggressive encounters, suggesting that risk of territory loss due to competition might favour increased boldness, antagonizing negative selective effects of predation on boldness [30]. However, although such studies suggest how competition may combine with co-stressors to influence selection, they do not document evolution in trait means or (co)variances, or resolve how personality versus plasticity might be involved.

#### (iii) Parasitism

Direct evidence that interplay between parasites and co-stressors mediates personality evolution is likewise rare. However, personality-dependent parasitism has been proposed to contribute to multi-stress trade-offs that could maintain personality variation. Bold-exploratory animals may use a wider array of habitats and resources, gaining access to resources but incurring parasite exposure [31,32]. For example, bold-exploratory grey squirrels (*Sciurus carolinensis*) were highly parasitized by gastrointestinal helminths [32]. Bold-aggressive animals might also incur higher parasite transmission through aggressive social interactions [31]. In vertebrates, elevated testosterone often proximally controls aggression [33] and suppresses immunity, which could increase parasitism [34]. Hence, a trade-off between parasitism versus resource limitation might support personality variation. However, some studies find no connection between personality and parasitism, as for risk-taking and malaria in collared flycatcher (*Ficedula albicollis*) [35], or suggest that personalities differ not in parasitism rate, but types of pathogens encountered [36]. Moreover, bold-aggressive behaviours that increase resource acquisition could dampen parasitism though resource-dependent improvement of immunity [25], and bolder animals could evolve better immunity, countering costs [31,37].

Parasites can also alter host behaviour through manipulating movement to elevate conspicuousness to predators or sickness behaviour [38]. Thus, differential exposure of personality types to parasites could generate positive or negative feedbacks that affect personality-dependent responses to co-stressors and selective pressure [39]. For example, greater exposure of bold individuals to parasites that upregulate movement could accentuate differences in depredation between bold and shy individuals, whereas the opposite might occur if shy individuals were more exposed.

High parasitism rates, not linked to personality, may also modify opportunity for selective responses to co-stressors by altering among-individual behavioural (co)variation and behavioural repeatability [40]. For example, the repeatability of amphipod (*Paracalliope flubiatilis*) phototaxis behaviour was reduced by microphallus parasites, as behaviour was homogenized [40], which could limit opportunity for selection. On the other hand, repeatability of periwinkle (*Littoraria irrorata*) antipredator behaviour was highest given simultaneous exposure to trematodes and predator cues [41].

Modelling and theoretical studies likewise suggest a role for parasites in multi-stress dynamics that may affect personality evolution [21,25]. A simulation model suggested that predation-starvation trade-offs alone could not maintain personality variation without temporal fluctuation in predation. However, given co-exposure to predation and parasites, personality variation was maintained without such fluctuation, but only if personalities were equally susceptible to parasitism [21]. Such models await empirical and mechanistic substantiation.

#### (iv) Contamination

Multi-stress effects involving contamination that may modify personality evolution could implicate various co-stressors. Bolder individuals may acquire resources at the expense of contamination. For instance, bolder zebrafish (*Danio rerio*) ingested more microplastics, but ingestion was reduced when brine shrimp abundance was high [42]. Moreover, contamination may impair antipredator behaviour or escape capacity, as in tropical fish (*Astyanax aeneus*) exposed to organophosphates [43]. Such effects could amplify negative selection on boldness under predation risk, especially if bolder individuals accumulate more contaminants [42]. However, evidence for personality-dependent contaminant exposure remains limited, and contamination does not always cause behavioural effects that could increase vulnerability to predators. For example, in gobies (*Bathygobius krefftii*), trophic transfer of contaminants from microplastics had no effect on activity and exploration behaviour [44].

Other co-stressors, including temperature and parasites, may also modify how contaminants affect selection on personality. High temperatures may elevate chemical toxicity, magnifying effects [45]. However, in European perch (*Perca fluviatilis*), pharmaceuticals and elevated temperature had independent, rather than interactive, effects on boldness and anxiety [46], illustrating that high temperature does not always amplify contaminant effects. Parasites could amplify contamination effects, if contamination increases susceptibility to infection, or absorb or metabolize contaminants, reducing effects [47]. However, for such interactions to affect selection on personality, contaminant exposure or sensitivity would have to be personality dependent, and few studies address this contingency.

As for parasitism, the effects of contamination on behavioural (co)variance could also alter selective effects of co-stressors on personality. In guppies (*Poecilia reticulata*), fluoxetine (Prozac) reduced among-individual variance in activity [48], which could limit selective or plastic responses to co-stressors.

#### (v) Temperature

Interactions involving a combination of temperature and various co-stressors may also affect personality evolution, but this contingency is underexplored. In ectotherms, increased temperature elevates metabolic rate (MR). Thus, given warming, ectotherms may be unable to forgo feeding to avoid predation or human disturbance factors, and a bolder personality may be promoted. For example, Atlantic cod (*Gadus morhua*) facing increased sea temperature emerged from refuges earlier, but on average did not increase feeding to compensate for MR, suggesting limited plasticity. However, among-individual variation suggested potential for selective response [49]. In endotherms, temperature may interact with resource limitation to mediate selection on personality, or plastic behavioural adjustments, by determining thermoregulatory costs. For instance, in hummingbirds, cold temperatures increase energy requirements such that territory value must be higher to make aggressive territory defence beneficial [50].

Furthermore, temperature may modify competition between species, through effects on MR or distributions, causing interactions between temperature and competition in mediating selection on aggression. Indeed, elevated temperature promoted aggression between invasive mosquito fish (*Gambusia holbrooki*) and Australian bass (*Macquaria novemaculeata*), whereas elevated salinity antagonized this effect. Hence, temperature and salinity could combine to determine frequency of agonistic interactions, and selection on personality variation [51].

#### (vi) Noise and light pollution

Also unexplored is the potential for the sensory pollutants of anthropogenic noise and artificial light at night (ALAN) to combine with co-stressors to alter selection on personality. For instance, noise can interfere with predator detection [52]. Thus, noise might synergistically interact with predation risk to promote shy-vigilant behaviour. However, increases in vigilance could accentuate predation-resource acquisition trade-offs, allowing for personality variation. Furthermore, noise can interfere with social cues, which might combine with resource availability to reshape social behaviour and behavioural trade-offs. Indeed, airport noise caused chiffchaffs (*Phylloscopus collybita*) to decrease song and increase physical aggression [53].

ALAN might interact with predation and resource limitation to impact selection on personality by affecting prey vulnerability to predators. The benefits of adjusting activity to ALAN are predicted to depend on predation-resource acquisition trade-offs. Thus, personality variation could be maintained given different solutions for balancing this trade-off. However, in nocturnal rodents, reductions in activity under ALAN were not related to an exploratory personality [54].

#### (vii) Human disturbance and urbanization

Human disturbance and urbanization have been proposed to favour bolder personalities, which could better maintain behaviour despite human disturbance, and quickly learn to exploit novel foods [55]. However, although a review found that urban–rural behavioural differences are widespread, the direction of differences is inconsistent [56]. Null relationships between urbanization and personality are also common, as in house sparrows (*Passer domesticus*) [57] and speckled wood butterflies (*Pararge aegeria*) [58].

Co-occurring stressors, notably predation risk, may modify whether human disturbance and urbanization select for bolder personalities, explaining such inconsistencies. By selecting for bolder personalities, frequent human disturbance, due to urbanization or ecotourism, might lead to maladaptation with respect to antipredator responses [59]. Hence, predation risk could counter increases in boldness within human-impacted habitats, as suggested by some evidence. For instance, invasive predators modified the association between boldness and urbanization in *Anolis* lizards and altered daily activity patterns [60]. Similarly, urban birds have elevated flight initiation distances near feral cat colonies, suggesting that predation may reduce evolutionary or plastic increases in boldness in urban animals [61].

Furthermore, rather than boldness generally declining with human disturbance, it might be reduced only in specific contexts. For instance, in five corvids and seven other bird species, urban birds were bolder towards litter but did not show general reductions in neophobia [62]. However, context-specific shifts in boldness may require costly increases in cognitive capacity [63]. Thus, pleiotropy driven by hormonal and genetic mechanisms might mediate general shifts in boldness-related personality traits in multi-stress contexts [63]. In lieu of specialized responses, general reductions in fearfulness could occur in environments encountering elevated human disturbance, limiting adaptive adjustments to co-stressors [63].

### (b) Multiple stressors may affect phenotypic expression of personality

Many examples discussed above grant insight not only into potential shifts in selection with multi-stress combinations, but also possible patterns of phenotypic plasticity. Thus, here, we do not again separately consider different stressors, but give selected examples to emphasize essential points.

#### (i) Behavioural reaction norms

**Figure 2.**
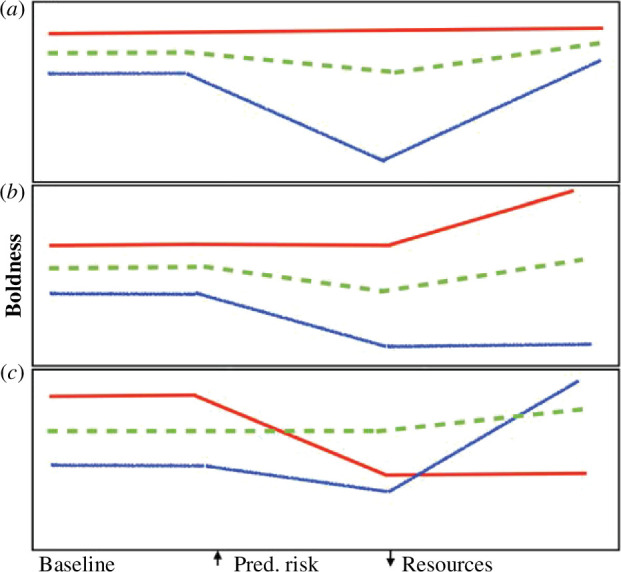
Hypothetical scenarios involving how different individuals (red = initially bold, green = intermediate, blue = shy) respond to sequential exposure to predation and resource limitation. In (*a*), response magnitude (boldness change) correlates with baseline boldness, with bolder individuals displaying minimal stressor responses (canalized reaction norms). In contrast, shyer individuals decrease boldness under predation risk, accentuating behavioural differences, but increase boldness under resource limitation, decreasing differences. Thus, behavioural variation is reduced under both stressors. In (*b*), bolder individuals decrease boldness less under predation risk, but increase boldness more under resource limitation. Hence, behavioural differences are most pronounced given two stressors. In (*c*), some individuals respond more strongly to predation and others to resources, with response magnitude uncorrelated with initial boldness. Personality ranking fluctuates between contexts, making among-individual variance changes difficult to predict.

Multiple stressors are expected to affect phenotypic expression as well as evolution of personality. Indeed, personality is shaped by genetics, environment and gene-by-environment interactions [64] and modified by state variables [16,20]. Adopting a behavioural reaction norm perspective helps elucidate how genetics and environment may combine to affect personality [65]. If reaction norms are parallel across environmental gradients, personality variation and rankings are maintained. However, given non-parallel reaction norms, personality variation and rankings may change across environments [65]. Given the variation in multiple stressors and differential individual responses to each, complex changes in personality variation and ranking may occur spatio-temporally, with different scenarios possible depending on how responses correlate with baseline (before stress) behaviour (figure 2).

#### (ii) Developmental stress

Multiple stressors experienced during development may strongly affect personality expression, altering fitness [66,67]. For example, during periods of intense lynx (*Lynx canadensis*) predation, snowshoe hares (*Lepus americanus*) show elevated cortisol, with resource limitation exacerbating effects. Cortisol is maternally transferred to offspring, causing offspring to have sensitized HPA axes [67]. Thus, developmental stress, caused by multi-stress exposure, may shape offspring stress sensitivity and behaviour. In selection lines of slow and fast exploring great tits, sibling competition and food shortage had different effects on the development of exploration behaviour depending on the selection line, suggesting the combined importance of genetics, epigenetics and multiple developmental stressors in determining phenotype [68]. Furthermore, in tadpoles (*Rana dalmatina*), activity risk-taking behavioural syndromes emerged given joint exposure to conspecifics and predator cues, likely due to among-individual differences in developmental behavioural plasticity [69]. However, more multi-stress studies are needed to determine the importance of multi-stress effects to personality development.

#### (iii) State dependency during adulthood

Multi-stress exposure that affects state variables (e.g. condition) may also affect personality expression. State-dependent models suggest that good condition can increase boldness by deeming animals better able to evade predators (state-dependent safety), promoting long-term personality stability through positive feedback [20]. As aforementioned, a model suggested that state-dependent safety should generate the most personality variation at intermediate combinations of predation and resources [20]. In contrast, asset protection-starvation avoidance, wherein individuals in better condition leave refuges later, generates negative feedback and may hence explain short- but not long-term among-individual behavioural differences [23,70,71]. However, the enhanced ability of individuals in good condition to gather resources due to better territories [72] or foraging efficiency [73], could counteract negative feedbacks. For example, young meerkats (*Suricata suricatta*) in good condition spent more time learning foraging skills and developed better foraging efficiency [73].

Multiple stressors could interact synergistically or antagonistically to affect state and personality expression. Starvation avoidance-asset protection may cause antagonistic interactions between resources and predation in determining risk-taking [23]. For instance, resource-limited snowshoe hares reduced foraging under predation risk less than food-supplemented counterparts [74]. In contrast, multiple energetic stressors (starvation and parasitism) might cause allostatic overload, threatening energy equilibrium [75] and causing synergistic behavioural changes. Furthermore, exposure to one stressor could impair coping with others, causing synergies. For example, contamination might reduce immunity, amplifying parasitism’s effects on individual state and behaviour [76]. However, as mentioned, some parasites may also uptake contaminants, potentially generating antagonistic interactions [47].

#### (iv) Physiological traits and state dependency

Variation in physiological traits with personality may influence how state dependency affects personality under multi-stress exposure. Boldness and activity have been associated with higher MR and pace-of-life [77,78]. Although some studies do not find this association [78,79], a recent meta-analysis found widespread, positive associations between MR and behaviours that either increase (e.g. foraging boldness) or cost (e.g. intense movement) net energy [78]. Individuals with higher MR deplete reserves faster, and may thus be less able to follow asset protection strategies, especially if resource limited or otherwise stressed. For instance, in sea bass (*Dicentrarchus labrax*), MR correlated with boldness towards predators only following food deprivation [80]. In seabass [81] and African sharptooth catfish (*Clarias gariepinus*) [82], relationships between MR and risk-taking increased under hypoxia. However, as a caveat, the above-described meta-analysis found no overall evidence that resource constraint modifies MR-behaviour relationships [78].

Interestingly, negative feedbacks might not prevent asset protection-starvation avoidance from contributing to long-term personality variation, if genetically shy-types have slow MR and thus better maintain reserves under food deprivation. Decreased resource intake can reduce MR [83], which could generate positive feedbacks on shyness-boldness when foraging under predation pressure. Indeed, a study in great tits found that perceived predation risk suppressed MR, but among-individual differences were not investigated [83].

## 4. Does personality affect responses to shifting multi-stress regimes?

### (a) Variation in behavioural and physiological responses

The behavioural reaction norm perspective implies that behavioural plasticity is itself an aspect of personality [66]. Hence, personality types may have different responses to shifting multi-stress landscapes. Multi-stress studies investigating this hypothesis remain rare. However, in great tits, exploratory personality predicted the proportion of time spent feeding on high- versus low-quality food as predation and starvation risk varied [84]. Some single stressor studies also suggest personality-dependent stressor responses. For example, slower exploring great tits took longer to resume feeding when exposed to traffic noise [85], shy and bold grasshoppers (*Melanoplus femurrubrum*) differed in foraging responses to sit-and-wait spider (*Pisaurina mira*) predators, causing personality-dependent mediation of trophic cascades [86], and reactive but not proactive Atlantic cod (*Gadua morhua*) reduced range size with rising sea temperature [87]. Other studies have identified among-individual differences in stressor responses, without making explicit connections to personality. For instance, some black-legged kittiwakes (*Rissa tridactyla*) increased heart rate by up to 15% when confronted with humans, whereas others showed little response [88]. However, many studies involving personality differences in stressor responses also report null results. For instance, studies involving disruption of sleep by traffic noise [89] and ALAN [90] in great tits found among-individual variation, but no difference among exploratory personalities. As aforementioned, effects of ALAN on nocturnal rodent behaviour were also unrelated to an exploratory personality [54].

Personality-dependent differences in stressor responses could be amplified or dampened given multi-stress exposure. Individuals may prioritize minimizing different stressors (e.g. predation versus resource limitation [84] when shifting behaviour in multi-stress contexts, with reaction norms hence diverging. Personality differences in stressor responses may also be amplified in multi-stress contexts if personality-dependent sensitivity or exposure to one stressor magnifies responses to others. For instance, in seabirds, reaching an energy threshold may trigger nest abandonment in disturbance-sensitive personalities nesting at human-frequented colonies, such that differential behavioural responses may emerge under resource stress [88]. Furthermore, personality types more exposed to parasites may fail to downregulate movement under increased predation risk [37], and contaminants (e.g. persistent organic pollutants [91]) may dysregulate physiological control pathways, altering responses to co-stressors. Hence, personality types more exposed to such stressors may show maladaptive multi-stress reaction norms, which diverge from responses of less exposed types.

On the other hand, exposure to one stressor may limit responses to co-stressors, causing reduction and convergence in responses. For example, manatees (*Trichechus manatus latirostris*) confronted with boat noise did not flee from thermal springs during winter, as avoiding hypothermia is essential [92,93], and pollutants can interfere with chemosensory predator detection, limiting antipredator responses [94]. Responding to one stressor may also reduce energy available to respond to others, which could diminish behavioural and physiological responses to co-stressors, and ultimately the number of concurrent stressors that organisms can tolerate [95]. In addition, as aforementioned, poor nutrition may decrease behavioural responsiveness towards humans and predators, as maintaining a minimum level of body reserves is critical [23]. Thus, behavioural insensitivity to disturbance could be incorrectly interpreted as indicating habituation or evolutionary increase in boldness, but actually reflect resource shortage [96]. However, meta-analytical evidence suggested no overall decrease in behavioural variance in boldness-related behaviours under nutritional stress, suggesting that behavioural responses were not always homogenized [23]. Such dynamics may limit animals’ capacities to adjust adaptively to multi-stress environments via plasticity, and impact evolutionary outcomes by affecting behavioural variance and population persistence.

### (b) Movement ecology and stressor avoidance/exposure

Personality differences in multi-stress responses may include differences in avoidance behaviour, mediated by movement ecology [10,95–97]. Personality types may partition habitat depending on risk of encountering stressors, with different individuals minimizing different stressors [96–98]. For instance, bold mud crabs (*Panopeus herbstii*) emigrated rapidly from poor quality reefs where predation was modest, avoiding resource limitation, but suffered higher predation than shyer crabs on high-quality reefs [96]. Furthermore, bolder dunnocks (*Prunella modularis*) settled in human-frequented areas [97], and bolder burrowing owls (*Athene cunicularia*) nested closer to roads [98]. However, some studies find that personality types are not differentially distributed across disturbance gradients [99]. Personality types could also differentially adjust daily activity to avoid stressors, partitioning temporal niches. A global-scale meta-analysis found that mammals have increased nocturnality by an average factor of 1.36 due to human disturbance [100]. Such effects may be more pronounced in risk-averse personalities, but this remains hypothetical.

Spatio-temporal avoidance may reduce stressor exposure of risk-averse personalities, elevating fitness, whereas bold types may fall into ecological traps by failing to avoid stressors. For example, American kestrels (*Falco sparverius*) often nest along roadways due to the presence of good foraging habitat, but show elevated corticosterone and nest abandonment, likely due to traffic noise [101]. However, avoiding one stressor may likewise have costs, by causing susceptibility to others. For instance, avoiding humans may elevate resource limitation and nutritional stress via lost opportunity costs [102].

Personality-linked differences in movement not associated with avoidance behaviour may also affect stressor exposure. Bold-exploratory individuals might encounter more diverse stressors over shorter timescales due to larger ranges, greater dispersal and invasion of new habitats [33,34]. Indeed, bolder wandering albatross (*Diomedea exulans*) foraged more widely [103], and exploratory lizards (*Lampropholis delicata*) dominate invasion fronts [104]. However, shy individuals do not always show restricted movement. In Cory’s shearwaters (Calonectris *borealis*), shy non-aggressive birds foraged farther from colonies under poor foraging conditions, likely because they were outcompeted for nearby resources [105].

The interplay between differential stressor avoidance and exposure, mediated through movement ecology, and differential physiological and behavioural responses to encountered stressors, may determine differences in physiological stress and fitness among personalities. If bolder animals incur greater multi-stress exposure, they might adjust responsiveness to avoid allostatic overload. Indeed, some evidence, for instance in song sparrows (*Melospiza melodia*) [106], suggests that animals in human-frequented habitats suppress HPA responses, counteracting chronic stress. However, a meta-analyses found no evidence that human disturbance consistently alters vertebrate stress physiology [107]. Alternatively, higher exposure of a personality type to given stressors could prevent coping with co-stressors, potentially generating positive feedbacks and selection against this personality type.

### (c) Cognition strategies

Cognitive strategies could also mediate how personality types respond to multiple stressors. Theorists and empirical studies have suggested that personality types have different cognitive strategies, with proactive individuals prioritizing speed over accuracy in learning, quickly solving novel problems, but being inflexible and slow to reversal learn [108–110]. For example, bolder marmosets (*Callithrix jacchus*) learned faster [109], whereas slower exploring black-capped chickadees (*Poecile atricapillus*) were quicker to reversal learn [110]. Personality types could also differ in specificity of stressor categorization during habituation. For instance, American crows (*Corvus brachyrhynchos*) recognize and classify specific humans as threatening [111]. The speed-accuracy trade-off hypothesis suggests that bolder individuals should more quickly categorize humans in general as non-threatening, discriminating less between individuals. Fast learning could allow bolder animals to exploit novel resources and quickly habituate to stressors [112,113]. However, lower accuracy and slower reversal learning could come at a cost, particularly in dynamic, multi-stress contexts.

However, a meta-analysis found that directions of relationships between personality and cognitive traits do not consistently coincide with the speed-accuracy trade-off hypothesis [114]. Thus, an alternative hypothesis is that personality types have context-dependent cognitive strengths, with cognitive diversity maintained within multi-stress contexts. The ecological intelligence hypothesis predicts that the cognitive strengths of species vary with ecology [115]. The same could apply for personalities within species. For instance, bolder individuals could quickly adopt novel resources, but fail to avoid new predators. Indeed, in generalist lizards (*Eutropis multifasciata*), faster explorers quickly learned to avoid unpalatable prey [116], but faster exploring convict cichlids (*Amatitlania nigrofasciata*) paid less attention to predators and responded to attack slowly [117]. Diversity in cognition and personality might hence be maintained, enhancing collective adjustment to changing multi-stress regimes [118].

Moreover, personality could influence how cognition is altered by stress exposure, with implications for behavioural and fitness outcomes. Negative effects of stressors on cognition have been demonstrated, although mild stress can potentiate cognition [119]. For instance, traffic noise reduced cognitive performance in zebra finches [120], and ALAN impaired inhibitory control in great tits, but effects were unrelated to exploratory personality [121]. Moreover, contaminants can impair cognition, even at low exposures [122]. Therefore, personality types more exposed or responsive to stressors could suffer greater cognitive and behavioural deficits in multi-stress landscapes. Personalities could also differ in the extent to which different stressors potentiate versus disrupt cognition, with optimal stress levels differing among individuals [123]. However, these hypotheses remain to be extensively tested.

## 5. Future research priorities

Our review has revealed many knowledge gaps that provide exciting future research directions. Here, we delineate research priorities with respect to major knowledge gaps.

### (a) Do multi-stress environments shape personality variation?

First, the ability to evaluate how multiple stressors affect personality evolution and phenotypic variation is limited by a deficit of multi-stress studies in general, and studies regarding specific stressors in particular. For instance, despite unprecedented climate change, relatively few studies consider how temperature may combine with co-stressors to affect selection on personality. More multi-stress studies are needed to explore which stressor combinations may synergistically, or antagonistically, affect personality evolution, including in the context of rapid environmental change.

Moreover, selection acts on trait variances, yet few studies report on variance components, limiting our capacity to predict evolutionary outcomes. Thus, examining how behavioural repeatability, and among- and within-individual variance, shift with multi-stress exposure will be critical to understanding effects of multi-stress environments on personality evolution [41].

### (b) Behavioural reaction norm and developmental perspectives

To understand joint contributions of genetics and plasticity in determining personality variation in multi-stress landscapes, more studies adopting reaction norm and developmental perspectives are also needed. Few studies have quantified behavioural reaction norms across multi-stress gradients. Moreover, despite extensive literature regarding how developmental stress can affect behavioural phenotypes [69], how multi-stress effects might differ from single-stressor effects has rarely been explored.

### (c) State dependency

Furthermore, state-dependent personality variation and state-behaviour feedbacks are proposed to have important effects on personality [16], but how multi-stress dynamics may affect state dependency remains little explored. Moreover, how physiological traits (e.g. MR) may mediate state-dependent personality within multi-stress contexts is an important outstanding question.

### (d) Personality-dependent multi-stress responses

With respect to personality-dependent stressor responses, our review highlighted that most studies exploring this issue involve single stressors. Thus, we advocate for studies that consider how among-individual differences in responses may be amplified or dampened in multi-stress contexts. Moreover, advances in biologging technologies [124] and tools for cognitive field studies [125] provide exciting opportunities to expand knowledge regarding how movement ecology and cognition may mediate personality-dependent multi-stress responses.

#### (i) Broader scale effects

Finally, personality variation within multi-stress landscapes might have broader scale implications. Personality variation might stabilize populations in changing multi-stress landscapes, given differential abilities to cope with stressors. Furthermore, given social interactions such as collective learning [126], diverse personalities might increase group performance in multi-stress contexts. However, filtering of personality types by disturbance regimes could also cause non-adaptive personality shifts and limit adaptation [127]. Changes in personality variation in response to multi-stress landscapes could have community- and ecosystem-level implications via changes in trophic interactions. Research focusing on fitness, community and ecosystem-level outcomes is needed to address these contingencies.

## 6. Conclusions

We have explored the hypothesis that personality variation may evolve through alternative strategies for balancing behavioural trade-offs within multi-stress landscapes, outlined how multiple stressors may modulate phenotypic personality variation and proposed that personality may affect responses to multi-stress regimes. Importantly, relative to single stressor viewpoints, a multiple stressor perspective has the potential to transform our understanding of the significance of animal personality variation in complex stress landscapes and under global change scenarios, as multi-stress interactions may reconfigure selective pressures on behavioural variation and because different personality types may be adapted to cope with different stressors or stressor suites. A multiple stressor perspective will be critical to elucidating how personality might affect among-individual differences in responses to anthropogenic environmental change, and forecasting implications for populations, communities and ecosystems. The growing multi-stress literature has yet to be thoroughly integrated with the behavioural ecology of personality. We hope that this review will stimulate this integration, exciting a productive body of research regarding the evolutionary ecology of animal personality within multi-stress landscapes.

## Data Availability

This article was not based on data collection. Hence, no datasets are associated with the paper. Electronic supplementary material clarifying review methodology and containing supplementary figures is available online [128].
